# Pharmaceuticals—Special Issue on Radiopharmaceutical Chemistry between Imaging and Endoradiotherapy

**DOI:** 10.3390/ph7070839

**Published:** 2014-07-16

**Authors:** Klaus Kopka

**Affiliations:** Division of Radiopharmaceutical Chemistry, Research Program Imaging and Radiooncology, German Cancer Research Center (dkfz), Im Neuenheimer Feld 280, D-69120 Heidelberg, Germany; E-Mail: k.kopka@dkfz-heidelberg.de

The fields of molecular biology, immunology and genetics have generated many important developments that advance the understanding of the induction and progression of oncological, cardiological and neurological diseases as well as the identification of disease-associated molecules and drugs that specifically target diseased cells during therapy. These insights have triggered the development of targeted radiopharmaceuticals which open up a new dimension of radiopharmaceutical sciences in nuclear medicine. Radiopharmaceuticals, also called radiotracers, are radiolabelled molecules, bearing a “radioactive lantern”, and used as molecular probes to address clinically relevant biological targets such as receptors, enzymes, transport systems and others. Positron emission tomography (PET) and single photon emission computed tomography (SPECT) realised in the *en-vogue* hybrid technologies PET/CT, SPECT/CT and PET/MRI represent the state-of-the-art diagnostic imaging technologies in nuclear medicine which are used to follow the trace of the administered radiopharmaceutical noninvasively thereby *in vivo* visualising and assessing biological processes at the subcellular and molecular level in a highly sensitive manner. In this connexion novel radiopharmaceuticals for the noninvasive molecular imaging of early disease states and monitoring of treatment responses *in vivo* by means of PET/CT, SPECT/CT and PET/MRI are indispensable prerequisites to further advance and strengthen the unique competence of radiopharmaceutical sciences. In the era of personalised medicine the diagnostic potential of *radiopharmaceuticals* is directly linked to a subsequent individual therapeutic approach called endoradiotherapy. Depending on the “radioactive lantern” (gamma or particle emitter) used for radiolabelling of the respective tracer molecule, the field of *Radiopharmaceutical Chemistry* can contribute to the set-up of an “*in vivo* theranostic” approach especially in tumour patients by offering tailor-made (radio)chemical entities labelled either with a diagnostic or a therapeutic radionuclide.

**Figure 1:**
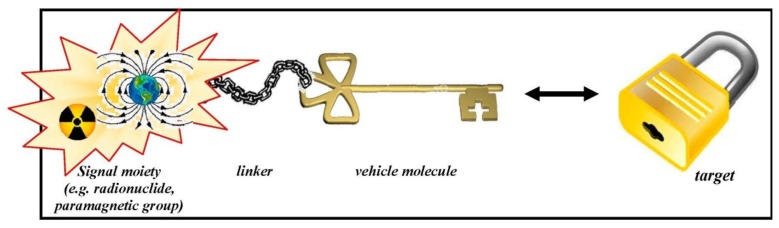
Schematic design of a molecular probe and its interaction with the target site. Data from [1].

Early-stage noninvasive molecular imaging enables to look at the biodistribution of molecular probes *in vivo* and facilitates to predict and monitor successful therapy strategies. The definition of “molecular probe imaging” is highlighted in detail in this special issue (Figure 1) [1]. Indeed the here presented special issue entitled *Radiopharmaceutical Chemistry between Imaging and Endoradiotherapy* intends to reflect many aspects of the general field of Radiopharmaceutical Sciences. The intention of the field radiopharmaceutical sciences is briefly summarised on the homepage of the Society of Radiopharmaceutical Sciences (SRS) : “At the heart of all nuclear imaging, radiopharmaceutical science is the design, synthesis and evaluation of compounds containing suitable radionuclides that can be used *in vivo* to trace (follow) a particular physiological or biochemical phenomena” [2].

In fact, according to reports of the United Nations Scientific Committee on the Effects of Atomic Radiation (UNSCEAR) more than 33 million diagnostic nuclear medicine examinations are performed annually worldwide. The annual frequency of diagnostic nuclear medicine examinations and of nuclear medicine treatments in health-care level I countries have thus increased tremendously, from 11 per 1,000 population in 1970–1979 to 19 per 1,000 in 1997–2007 and for therapeutic nuclear medicine procedures from 0.17 per 1,000 population in 1991–1996 to 0.47 per 1,000 in 1997–2007 [3]. These data are consistent with the trend towards the increasing relevance of diagnostic and therapeutic applications using corresponding radiopharmaceuticals in the respective health care systems. These numbers also illustrate the high demand to develop new diagnostic and therapeutic radiopharmaceuticals in translational projects in order to invest in modern personalised patient-centered health care using nuclear medicine technologies.

To succeed in the design of targeted high-affinity radiopharmaceuticals that can measure the alteration of a respective biological target several aspects need to be considered:

(i) reasonable pharmacokinetic behaviour adjusted to the physical half-life of the used radionuclide
(ii) ability to penetrate and cross biological membranes and permeability barriers
(iii) usage of chemical as well as biological amplification strategies (e.g., pretargeting, biological trapping of converted ligands, change of the physicochemical behaviour of the radiopharmaceutical after target interaction and combination with biotransporters)
(iv) availability of radiopharmaceuticals with high specific activities and *in vivo* stability

**Figure 2:**
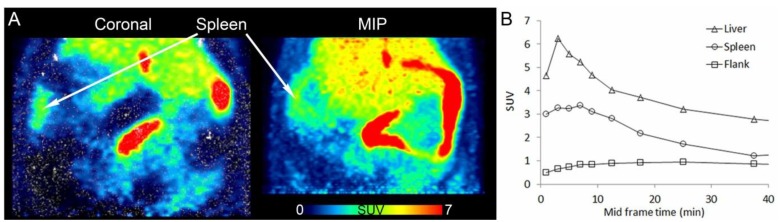
PET/CT images of [^11^C]KP23 in the spleen-liver region in rat. (**A**) Coronal section and maximal intensity projection (MIP) averaged from 6 to 15 min p.i. (**B**) TACs of [^11^C]KP23 in spleen, liver and flank. Data from [4].

**Scheme 1:**
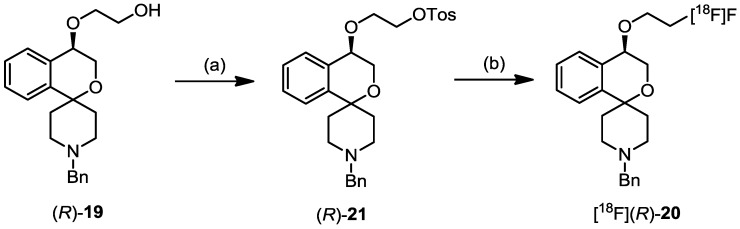
Radiosynthesis of [^18^F]-**(*R*)**-**20**. Data from [5].

Two approaches are presented in this special issue that aim at reaching neurologically relevant receptors in the central nervous system which are confronted with the penetration of the blood-brain barrier, the key example of a selective permeability barrier, formed by capillary endothelial cells connected by tight junctions. Mu L *et al.* have started a project on targeting the cannabinoid receptor subtype 2 (CB2) with a promising ^11^C-labelled 2-oxoquinoline derivative [^11^C]KP23. The CB2 receptor has been shown to be up-regulated in activated microglia and therefore plays an important role in neuroinflammatory and neurodegenerative diseases such as multiple sclerosis, amyotrophic lateral sclerosis and Alzheimer’s disease (Figure 2) [4]. Holl K *et al.* synthesised new enantiomers of ^18^F-labelled 4-substituted spirocyclic 2-benzopyrans and investigated their affinity towards σ receptors. PET tracers, which are able to label selectively σ1 receptors, are of high interest for the diagnosis of diseases such as Alzheimer’s Disease, neuropathic pain, schizophrenia and major depression in which the σ1 receptor obviously is involved (Scheme 1) [5].

*Reagents and Conditions*: (**a**) p-TosCl, NEt_3_, 4-DMAP, CH_2_Cl_2_, RT, overnight, 46%; **(*S*)-21** was obtained in 32% yield starting with **(*S*)-19**. (**b**) K[^18^F]F, Kryptofix K222, acetonitrile, 82 °C, 20 min.

**Scheme 2:**
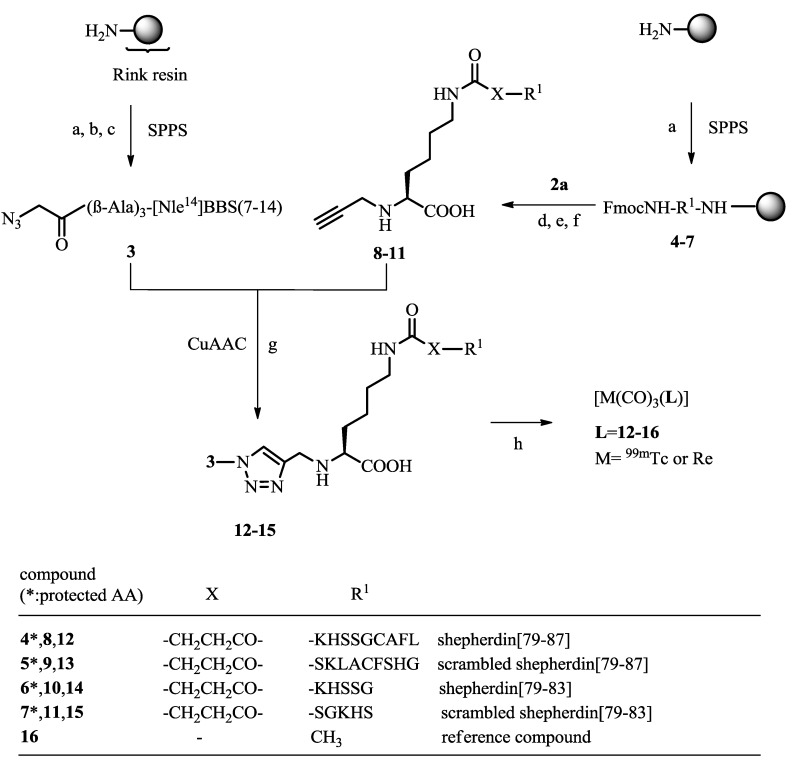
Synthesis of peptides and assembly of (radio)metal-labeled, multifunctional conjugates. Data from [6].

An example of how a radiotracer could be entrapped after binding to the chosen receptor is given by Fischer CA *et al.* with the contribution *A Bombesin-Shepherdin Radioconjugate Designed for Combined Extra- and Intracellular Targeting*. Unfortunately, while the specificity of the radioconjugates towards the gastrin-releasing peptide receptor (GRPr) could be confirmed, the cellular externalisation of the radioactivity was not improved (Scheme 2) [6].

(a) SPPS: (i) piperidine in DMF; (ii) Fmoc-amino acids, TBTU-HOBt, (b) azidoacetic acid, HATU; (c) cleavage solution: trifluoroacetic acid, phenol, water, triisopropylsilane (87.5/5/5/2.5%), 2–5 h, rt; (d) SPPS: **2a**, HATU, (e) cleavage solution, 2–8 h, rt; (f) LiOH (0.5 M), 1–3 h, rt; (g) CuAAC in solution; CuSO_4_ (0.2 M), Na-ascorbate (0.4 M), 1 h, rt; (h) (radio)metal labeling; for M=^99m^Tc: **12**–**16** (0.1 mM, aq.), [^99m^Tc(CO)_3_(H_2_O)_3_]^+^, 100 °C, 30 min; for Re complexes: **12**–**16** (0.4 mM, aq.), [Et_4_N]_2_[Re(CO)_3_(Br)_3_], 100 °C, 60 min; AA = amino acid.

**Figure 3:**
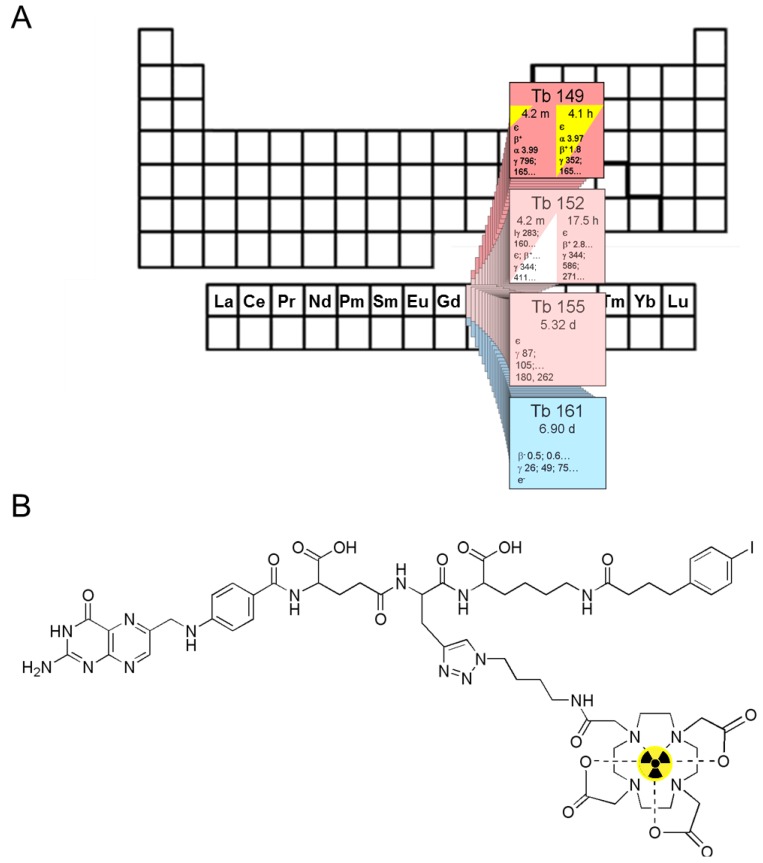
(**A**) The α-particle-emitting ^149^Tb as one of four medically interesting terbium isotopes which belong to the series of chemical elements called lanthanides (15 elements from La to Lu); (**B**) Chemical structure of the radiolabeled DOTA-folate conjugate (cm09) with an albumin binding entity (speculative coordination sphere of the Tb-DOTA-complex). Data from [7].

Ideally, the molecular structure of the tailor-made precursor compound designed for radiolabelling should allow attaching either gamma emitters (e.g., iodine-123) and positron emitters (e.g., iodine-124) or particle emitters (e.g., iodine-131), respectively. This results either in diagnostic radiopharmaceuticals for SPECT/CT (^123^I-labelled radiotracers), PET/CT and PET/MRI (^124^I-labelled radiotracers, respectively), or in a therapeutic radiopharmaceutical (then labelled with ^131^I) for targeted systemic radionuclide therapy (endoradiotherapy) with identical chemical structure. Similarly to the high efficiency of the combination of radiological diagnostics and radiation therapy, this combined *in vivo* theranostic approach with targeted radiotracers provide the potential to significantly improve the management of many diseases and the application of individually adapted therapy strategies. One contribution at the current basic research front on *Folate Receptor Targeted α-Therapy using Terbium-149* addresses the mentioned *in vivo* theranostic approach and is described by Müller C *et al.* who discuss the potential matching pairs of diagnostic and therapeutic folate receptor (FR)-targeted radiotracers bearing different radioisotopes of terbium (Figure 3) [7].

**Figure 4:**
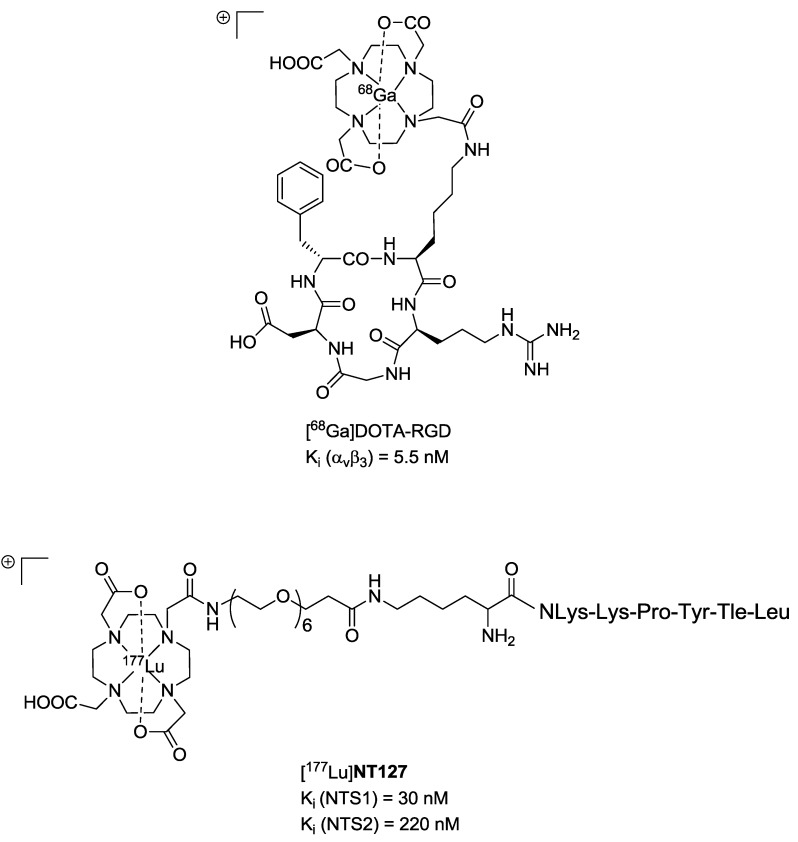
Chemical structures of [^68^Ga]DOTA-RGD [10] (**top**) and [^177^Lu]NT127 (**bottom**). Data from [8].

**Figure 5:**
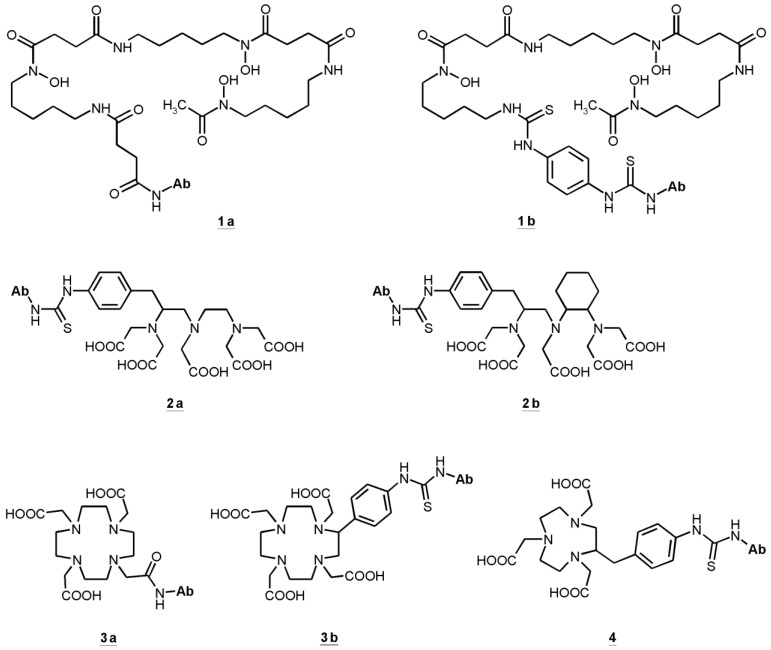
Bifunctional chelators (BFC) used in cetuximab conjugates: succinylated desferoxamine (*N*-sucDf, **1a**), desferoxamine-p-SCN (Df–Bz–NCS, **1b**), p-SCN-Bn-DTPA (**2a**), CHX-A''-DTPA (**2b**), DOTA-NHS-ester (**3a**), p-SCN-Bn-DOTA (**3b**), p-SCN-Bn-NOTA (**4**). Data from [9].

The neurotensin receptor (NTS1) is an attractive biological target for the molecular imaging and endoradiotherapy of NTS-positive tumours owing to the overexpression in a range of malignancies. One contribution shows an example of a ^177^Lu-labelled NTS1 radioligand for endoradiotherapy of a preclinical colon tumour model and subsequent imaging of successful therapy by μPET using [^68^Ga]Ga-DOTA-RGD as a specific radiotracer for imaging angiogenesis (Figure 4) [8]. Another attractive receptor which is important for the progression of cancer represents the epidermal growth factor receptor (EGFR). In immunotherapy the anti-EGFR-antibody Cetuximab (Erbitux^®^) is used for the treatment of different tumours. EGFR can thus be considered as an ideal biological target for combinatorial diagnostic and therapeutic approaches using corresponding radiolabelled cetuximab conjugates. Here Sihver W *et al.* summarises the hitherto developed radiolabelled cetuximab conjugates for EGFR-targeted diagnosis and therapy (Figure 5) [9].

**Figure 6:**
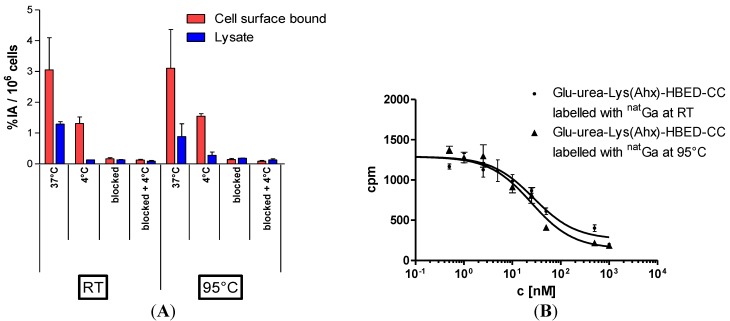
(**A**) LNCaP-cell binding and internalisation of [^68^Ga]Ga-PSMA-HBED-CC labelled at RT or 95 °C, respectively. Specific cell uptake was determined by competitive blockade with 500 µM of the PSMA inhibitor 2-PMPA. Values are expressed as percentage of applied radioactivity bound to 10^6^ cells [%IA/10^6^ cells]. Data are expressed as mean ± SD (*n* = 3); (**B**) Determination of binding affinity of [^nat^Ga]Ga-PSMA-HBED-CC to LNCaP cells as a function of the labelling temperature. The cells (10^5^ per well) were incubated with the radioligand (^68^Ga-labelled Glu-urea-Lys(Ahx)-HBED-CC) in the presence of different concentrations of ^nat^Ga-analyte (0–5000 nM, 100 µL/well). Data from [11].

**Scheme 3:**
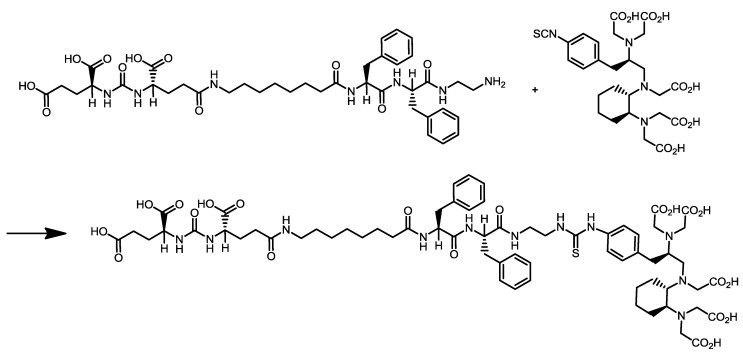
Synthesis of cyclohexyldiethylenetriamine pentaacetic acid (5*S*,8*S*,22*S*,26*S*)-1-amino-5,8-dibenzyl-4,7,10,19,24-pentaoxo-3,6,9,18,23,25-hexaazaoctacosane-22,26,28-tri-carboxylic acid trifluoroacetate (CHX-A''-DTPA-DUPA-Pep). Data from [12].

The highly sensitive visualisation of prostate cancer by PET/CT imaging of the prostate-specific membrane antigen (PSMA) has gained highest clinical impact during recent years. The new PET radioligand [^68^Ga]Ga-PSMA-HBED-CC was developed at the DKFZ and is the most promising PET tracer for the imaging of prostate cancer to date and clinical trials are currently going to be initiated. Therefore, novel important preclinical data of [^68^Ga]Ga-PSMA-HBED-CC and relevant aspects of its radiopharmaceutical good manufacturing practice (GMP)-compliant production are presented in this special issue (Figure 6) [11]. One additional representative PSMA inhibitor, CHX-A''-DTPA-DUPA-Pep, potentially applicable both as diagnostic and therapeutic radiopharmaceutical was radiolabelled with the PET radionuclide gallium-68 (^68^Ga) as well as with the beta-minus particle emitters yttrium-90 (^90^Y) and lutetium-177 (^177^Lu) which are clinically relevant for systemic radionuclide therapy (*i.e.*, endoradiotherapy) (Scheme 3) [12]. 

**Figure 7:**
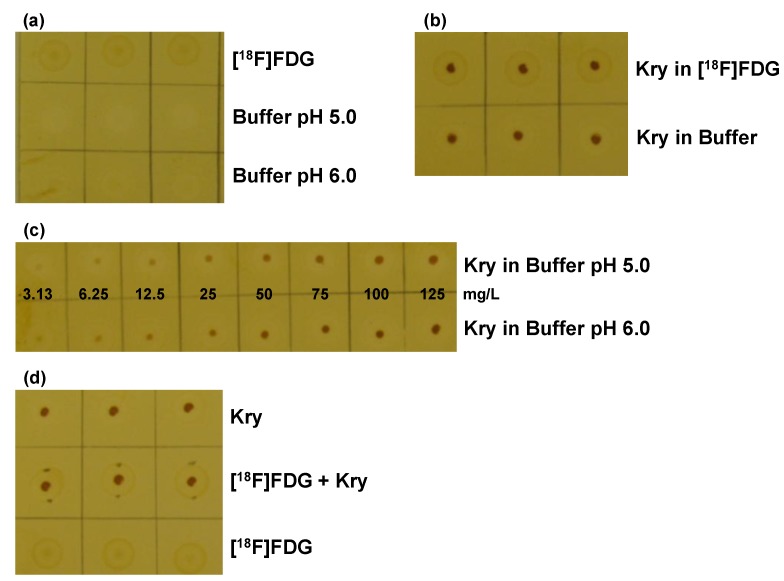
Validation of the TLC spot test for Kry in [^18^F]FDG: **(a)** Test for specificity. TLC plate after iodine staining, samples (*n* = 3) are without Kry. 1. row: [^18^F]FDG solution; 2. row: citrate matrix buffer (pH 5.0); 3. row: citrate matrix buffer (pH 6.0); **(b)** Test for Specificity. Samples (*n* = 3) of 100 mg/L Kry in either [^18^F]FDG solution (1. row) or citrate buffer matrix (pH 6.0; 2. row); **(c)** Test for specificity and detection limit. Samples of Kry standard solutions with concentrations of 3.13, 6.25, 12.5, 25, 50, 75, 100 and 125 mg/L in buffer matrix (from left to right; 1. row: pH 5.0; 2. row: pH 6.0; *n* = 3; one example shown); **(d)** Test for Accuracy. Comparison of samples (*n* = 3) of 100 mg/L Kry in matrix (pH 5.0; 1. row); [^18^F]FDG solution and 100 mg/L Kry added (pH 5.0; 2. row) and pure [^18^F]FDG solution (3. row). Data from [14].

The GMP-compliant production and quality control of radiopharmaceuticals have to conform to all aspects of the relevant EU-GMP regulations and radiation protection rules. Specific guidance on current good radiopharmacy practice (cGRPP) for the small-scale preparation of radiopharmaceuticals has been recently published by the EANM radiopharmacy committee [13]. One representative contribution in this special issue deals with the necessary quality control equipment on-site and highlights an improved example of how the analysis of residual contaminations (here: phase transfer catalysts) in radiopharmaceutical formulations can be simply realised by a rapid thin layer chromatography (TLC) spot test which is adapted to the current monographs of the EU and US Pharmacopoeia (Figure 7) [14].

**Figure 8:**
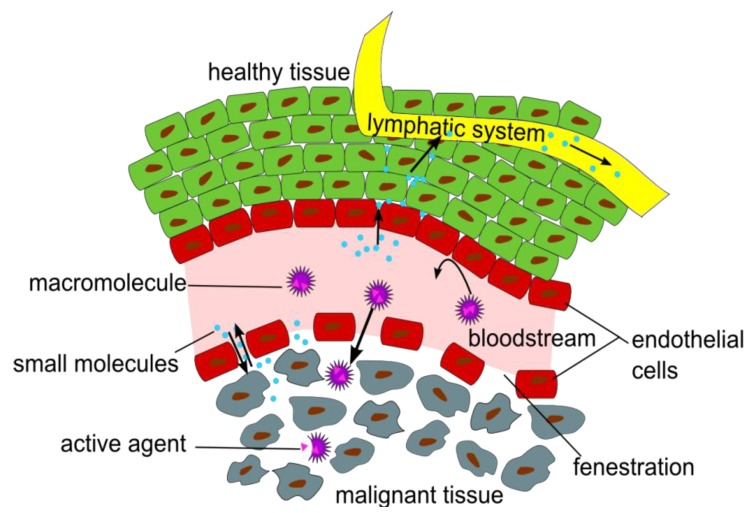
Illustration of the Enhanced Permeation and Retention (EPR) effect of macromolecular structures as drug delivery systems in malignant tissue. Data from [15].

Finally, one contribution gives an overview of radiolabelled nanoparticles and polymers for PET imaging. Nanomedicine has attracted high interest over the last decades as nanoparticles (NPs) have been designed and used for various purposes, such as magnetic resonance imaging (MRI), computed tomography (CT) and optical imaging (OI) or simply for improved drug delivery. The question is: What is the ideal nanodimensional architecture usable as drug delivery system or for multimodality imaging by means of PET/CT and PET/MRI? In detail the review article of Stockhofe *et al.* provides information about radiolabelling procedures of NPs or polymers intended for PET imaging and their potential use as drug delivery systems (Figure 8) [15]. A complementary editorial recently published elsewhere tries to give some answers where to use nanotechnology implemented in radiopharmaceuticals [16].

In summary this comprehensive special issue *Radiopharmaceutical Chemistry Between Imaging and Endoradiotherapy* covers a broad spectrum of representative internationally recognized research and development projects and nicely shows the complexity of the multidisciplinary field radiopharmaceutical sciences located in between nuclear chemistry, radiochemistry, organic chemistry, bioorganic and -inorganic chemistry, labelling chemistry, medicinal chemistry, radiopharmacology and nuclear medicine. The special issue published in *Pharmaceuticals* intends to reach and attract rather extended readership including students, PhD students and young scientists and talents from medicinal disciplines such as nuclear medicine, radiology and radiation therapy, but especially from natural sciences such as chemistry, pharmacy, biotechnology, technical engineering and physics who are looking for highly innovative translational research conceptions in radiopharmaceutical sciences.
